# Does age matter? The impact of rodent age on study outcomes

**DOI:** 10.1177/0023677216653984

**Published:** 2016-06-15

**Authors:** Samuel J Jackson, Nick Andrews, Doug Ball, Ilaria Bellantuono, James Gray, Lamia Hachoumi, Alan Holmes, Judy Latcham, Anja Petrie, Paul Potter, Andrew Rice, Alison Ritchie, Michelle Stewart, Carol Strepka, Mark Yeoman, Kathryn Chapman

**Affiliations:** 1 National Centre for the Replacement, Refinement and Reduction of Animals in Research, London, UK; 2 Division of Neurology, Kirby Center for Neurobiology, Boston Children’s Hospital, Boston, US; 3 Immunoinflammation TAU, GlaxoSmithKline, Stevenage, UK; 4 Centre for Integrated Research into Musculoskeletal Ageing, University of Sheffield, Sheffield, UK; 5 Pharmacy and Biomolecular Sciences, University of Brighton, Brighton, UK; 6 Centre for Rheumatology, UCL Division of Medicine, Royal Free Campus, London, UK; 7 Laboratory Animal Science, GlaxoSmithKline, Stevenage, UK; 8 Rowett Institute of Nutrition & Health, University of Aberdeen, Aberdeen, UK; 9 Disease Models and Translation, Mammalian Genetics Unit, MRC Harwell, Harwell, UK; 10 Pain Research, Department of Surgery & Cancer, Imperial College London, London, UK; 11 Division of Cancer & Stem Cells, University of Nottingham, Nottingham, UK; 12 The Mary Lyon Centre, MRC Harwell, Harwell, UK; 13 Preclinical Services, Charles River, Edinburgh, UK

**Keywords:** rodent, age, experimental design, development, senescence

## Abstract

Rodent models produce data which underpin biomedical research and non-clinical drug trials, but translation from rodents into successful clinical outcomes is often lacking. There is a growing body of evidence showing that improving experimental design is key to improving the predictive nature of rodent studies and reducing the number of animals used in research. Age, one important factor in experimental design, is often poorly reported and can be overlooked. The authors conducted a survey to assess the age used for a range of models, and the reasoning for age choice. From 297 respondents providing 611 responses, researchers reported using rodents most often in the 6–20 week age range regardless of the biology being studied. The age referred to as ‘adult’ by respondents varied between six and 20 weeks. Practical reasons for the choice of rodent age were frequently given, with increased cost associated with using older animals and maintenance of historical data comparability being two important limiting factors. These results highlight that choice of age is inconsistent across the research community and often not based on the development or cellular ageing of the system being studied. This could potentially result in decreased scientific validity and increased experimental variability. In some cases the use of older animals may be beneficial. Increased scientific rigour in the choice of the age of rodent may increase the translation of rodent models to humans.

## Résumé

Les modèles murins produisent des données qui sous-tendent la recherche biomédicale ainsi que les essais non-cliniques de médicaments, mais il est rare que les données issues des rongeurs se transforment en résultats cliniques réussis. Il existe un nombre croissant de preuves montrant que l’amélioration de la conception expérimentale est essentielle à l’amélioration de la nature prédictive des études menées sur des rongeurs, ainsi qu’à la réduction du nombre d’animaux utilisés en recherche. L’âge, un facteur important dans la conception expérimentale, est souvent mal pris en compte dans les rapports et peut être négligé. Les auteurs ont mené une enquête pour évaluer l’âge utilisé dans une gamme de modèles, et le raisonnement motivant le choix de l’âge. D’après les 297 répondants fournissant 611 réponses, les chercheurs ont constaté que les rongeurs utilisés avaient le plus souvent de 6 à 20 semaines d’âge, indépendamment de la biologie faisant l’objet de l’étude. L’âge que les répondants estimaient « adulte » variait entre 6 et 20 semaines. Des raisons pratiques justifiant le choix de l’âge des rongeurs ont été fréquemment avancées, l’augmentation des coûts liés à l’utilisation d’animaux plus âgés et le maintien de la comparabilité des données historiques étant deux importants facteurs limitatifs. Ces résultats mettent en lumière que le choix de l’âge n’est pas uniforme au sein de la communauté scientifique et qu’il est rarement fondé sur le développement ou le vieillissement cellulaire du système étudié. Cela pourrait entraîner une diminution de la validité scientifique et une augmentation de la variabilité expérimentale. Dans certains cas, l’utilisation d’animaux plus âgés peut s’avérer bénéfique. La rigueur scientifique accrue dans le choix de l’âge des rongeurs peut augmenter l’application de modèles de rongeurs à l’homme.

## Abstract (de)

Nagermodelle liefern Daten zur Untermauerung biomedizinischer Forschung und nicht-klinischer Arzneimittelprüfungen, doch an einer Übertragung von mit Nagern erzielten Resultaten in erfolgreiche klinische Ergebnisse mangelt es häufig. Es gibt zunehmend Belege dafür, dass die Verbesserung der Versuchsgestaltung der Schlüssel für bessere vorausschauende Aussagen von Nagerstudien und für eine Senkung der Zahl von Versuchstieren ist. Das Alter, ein wichtiger Faktor für die Versuchsgestaltung, wird oft ungenügend vermerkt und beachtet. Die Autoren führten eine Befragung zur Bewertung des Alters von bei verschiedenen Modellen verwendeten Tieren und die Gründe für die jeweilige Altersentscheidung durch. Dabei gaben 297 Befragte 611 Antworten. Die Forscher berichteten, dass sie meist Nager im Altersbereich von 6 bis 20 Wochen verwendeten, unabhängig vom zu untersuchenden biologischen Gegenstand. Das von Befragten als ,,adult“ bezeichnete Alter variierte zwischen sechs und 20 Wochen. Für die Wahl des Nageralters wurden häufig praktische Gründe angeführt, wobei erhöhte Kosten bei der Nutzung älterer Tiere und die Vergleichbarkeit historischer Daten als zwei hemmende Faktoren genannt wurden. Diese Ergebnisse zeigen, dass Forscher keine einheitlichen Kriterien bei der Wahl des Alters anlegen und dass ihre Wahl häufig nicht auf der Entwicklung oder Zellalterung des untersuchten Systems basiert. Daraus könnte potenziell eine reduzierte wissenschaftliche Gültigkeit und erhöhte experimentelle Variabilität resultieren. In manchen Fällen kann u. U. die Verwendung älterer Tiere von Vorteil sein. Erhöhte wissenschaftliche Stringenz bei der Wahl des Alters von Nagern kann u. U. der Übertragung von Nagermodellen auf Menschen förderlich sein.

## Resumen

Los modelos de ratones producen datos que respaldan la investigación biomédica y pruebas de fármacos no clínicas, pero muchas veces falta la conversión de roedores a unos resultados clínicos fructíferos. Existe cada vez más evidencia de que la mejora del diseño experimental es clave para mejorar la naturaleza predictiva de los estudios con roedores y para reducir el número de animales utilizado en estudios de investigación. La edad, un factor importante en el diseño experimental, no se registra adecuadamente con frecuencia y puede pasarse por alto. Los autores llevaron a cabo un estudio para evaluar la edad utilizada para una serie de modelos y el razonamiento detrás de la elección de distintas edades. De 297 participantes que aportaron 611 respuestas, los investigadores registraron un uso frecuente de roedores de entre 6 y 20 semanas de edad, independientemente de su estudio biológico. La edad considerada como "adulta" por parte de los participantes variaba de entre 6 y 20 semanas. Con frecuencia se registró que la elección de las edades de los roedores se debe a motivos prácticos, con un aumento del coste asociado con el uso de animales mayores y el mantenimiento de la comparabilidad de datos históricos siendo dos factores decisivos. Estos resultados destacaron que la elección de edad es inconsistente en la comunidad de investigadores y a menudo no se basa en el desarrollo o el envejecimiento celular del sistema estudiado. Esto potencialmente podría provocar una reducción de la validez científica y un aumento de la variabilidad experimental. En algunos casos, el uso de animales mayores podría ser beneficioso. Un mayor rigor científico en la elección de la edad de los roedores podría aumentar la conversión de modelos de roedores a humanos.

The most frequently used mammals in scientific research are rodents, predominantly rats and mice. There are various experimental factors which are critical to the quality of data obtained from rodent models and these factors differ according to the requirements of the experiment.^1^ Previous studies have shown that reporting of animal experiments in peer-reviewed publications is poor due to omission of details of experimental design such as blinding, randomization, and details of strain, sex and age.^2,3^

Significant differences exist in disease-relevant systems in young or aged animals compared with middle-aged counterparts, and these differences may affect the outcome of studies investigating basic disease biology, mechanisms of drug action and efficacy. Inconsistent choice of age in rodent models between experiments or laboratories has the potential to impact on data quality, potentially increasing variability and reducing relevance to the human disease being studied. In basic or mechanistic studies, biological pathways or mechanisms of action may be obscured by inter-animal variability arising from inappropriately aged animals. In drug development, the translation of compounds from non-clinical research to phase II clinical trials has been shown to be poor in many therapeutic areas, with a lack of predictive rodent models for human disease partly responsible for attrition.^4,5^ Use of a suboptimal and variable age of animal could be contributing to this.

The age at which animal models are commonly used is 8–12 weeks. In this age range, many developmental processes are ongoing,^6–11^ and changes in physiology with age may have a large impact on experimental variables. For example:

- Peak bone mass is not reached until around 26 weeks of age in rodents.^7,12,13^
- The development of the immune system is defined by changes in thymus size and cellular content over early development^14,15^ as well as key immunological markers.^16^ It has been shown that B-cells have an immature phenotype until four weeks of age,^17^ T-cell responses mature around eight weeks of age,^18^ and T and B-lymphocyte production increases over the first 26 weeks of life.^19^
- Significant brain growth is ongoing in the rat until nine weeks of age^6^ and central nervous system myelination in limbic structures is not complete until six weeks of age,^10^ development of mouse spinal cord, hippocampus and olfactory structures is ongoing until 11 weeks of age.^11^

Additionally, older animals entering senescence may respond differently to their younger counterparts.^13,20–22^ For example:

- Factors such as menopause and old age are risk factors for bone diseases in humans, but are poorly modelled in younger rodents and require careful consideration of translational biomarkers in older rodents to ensure cross-species comparability.^23–25^
- The profound effect of age in models of stroke has been documented,^26–28^ and young animals have been used to model a disease generally seen in older humans. Alterations in blood flow and brain biochemistry with age have a significant impact in humans, but these pathologies are not reflected when modelling the disease with young rodents. Older rats have been shown to exhibit a difference in susceptibility to,^28^ or take longer to recover from,^29^ ischemic insults.
- Age-related phenotypes are being uncovered in commonly used inbred strains. For example, the widely used C57BL/6J mouse has impaired glucose tolerance due to a missense mutation in nicotinamide nucleotide transhydrogenase (*Nnt*),^30^ which has implications for diabetic phenotypes, as well as causing age-related decline in mitochondrial functions.^31,32^ In addition C57BL/6J mice have a mutation in the cadherin 23 (*Cdh23*) gene, resulting in age-related hearing loss.^33^ This may impact on behavioural studies as the mice age.
- The microbiome plays important roles in the maintenance of appropriate immune system function and response to disease. Ageing can affect the composition of the mouse microbiome,^34^ and this has been shown to modulate mouse models of allergic airway disease,^35^ potentially reducing the quality of data obtained.
- Drug metabolism by the liver has a critical impact on systemically administered compounds, and is therefore of primary importance during the development of new pharmaceuticals. The gene expression of critical liver enzymes is dramatically different between young and older counterparts,^36,37^ potentially leading to discrepancies or errors affecting drug candidate selection or development.

A working group was convened by the National Centre for the Replacement, Refinement and Reduction of Animals in Research (NC3Rs) to explore the age of rodents used to model physiology, disease or drug efficacy, and to assess the impact on scientific outcomes, with the aim of improving selection of the age of rodent models. Here, we present literature examples illustrating the importance of a well-informed age choice, and data from a survey regarding the age of rodents used in biomedical research. In addition, two data-based case studies are included as supplemental data (Figures S2 and S3; all supplemental material can be found online at http://lan.sagepub.com). By improving decision-making around the age of rodent used in biomedical research, the number of animals used can be reduced while maintaining or improving data quality.

## Methods

### Survey regarding age of rodent used

A cross-sector working group, reflected in the authorship, was formed from a diverse range of disciplines with an interest in the impact of age on rodent models of disease. In order to explore the age at which rodents are currently being used in research, an online survey was formulated by the expert working group with reference to published guidance on survey design.^38^ The survey requested data on the type of rodent model used by the researcher, and the age chosen for the model (see supplemental Table S1). Researchers could enter information on up to five separate models. Researchers were also asked for additional information on why the specific age was chosen and evidence for age suitability of the model. A drop-down list was used to establish reasons for choice of age (see supplemental Table S1 Q4).

The survey was pilot-tested and refined within the working group, and distributed as a hyperlink in a standard text via 20 learned societies and research charities, as well as individual scientists and scientific networks. The disciplines covered cancer, cardiovascular disease, endocrinology, immunology, musculoskeletal disease, neuroscience, and respiratory disease, and encompassed academic and pharmaceutical researchers in the UK and EU. This enabled distribution to a representative sample of researchers, as described in the Results section.

The data analysis workflow is shown in supplemental Figure S1. Data from the survey was anonymized and initially analysed to assess information about respondent location and field of work, and which models were represented in the data-set. Models which could not be classified (see below) or without a justification for the age used were removed. Each model was individually classified by ‘System/Process’ (see supplemental Table S2) and ‘Disease/Condition’ (a list of 233 diseases and conditions) as defined by the Wellcome Trust (London, UK).^39^

#### Comparison of age used for the same model paradigm across laboratories

Where the same model paradigm was reported in multiple responses, these were grouped and the range of ages plotted.

#### Age considered ‘adult’

Where the choice of age was justified by describing the animal as ‘adult’, the range of ages was plotted as a frequency graph.

## Results

Given the literature demonstrations of changes in disease modelling in rodents with age, a survey was designed to collect information on the age at which researchers used rodents to model human disease or physiology. A diagrammatic description of the data workflow followed in this publication can be found in supplemental Figure S1.

There were 297 respondents to the survey, predominantly from the academic sector (80%), with 18% from the pharmaceutical sector. Location information was provided by 108 respondents (35% of the total) which demonstrated that responses were received from 27 UK and 31 non-UK locations.

The survey allowed for up to four responses per respondent. The total number of responses received was 611, representing a cross section of disciplines including neuroscience, immunology, cancer, genetics, physiology and toxicology. The range of ages reported was wide (2–160 weeks) but was heavily clustered around the 8–12 week age range for both mice and rats.

**Figure 1.**
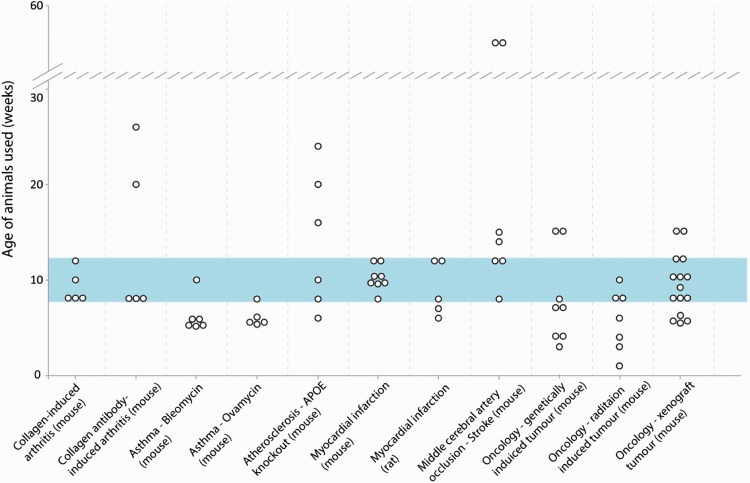
Age ranges of rodent reported for specific models. Where a model was described sufficiently, there was opportunity to compare the age of specific model systems across responses. Respondents described that a range of ages of rodent were used for a given model. Ages used for all models clustered around the 8–12 week range, regardless of the biology being studied. The use of some ages, particularly at the low or high extremes, was justified by a specific biological reasoning.

When the same model paradigm was reported multiple times, comparison of the age used across different laboratories was possible. Figure 1 illustrates the range of ages used for each model. This demonstrates that researchers in different laboratories used different ages of animal in the same experimental paradigm.

**Figure 2.**
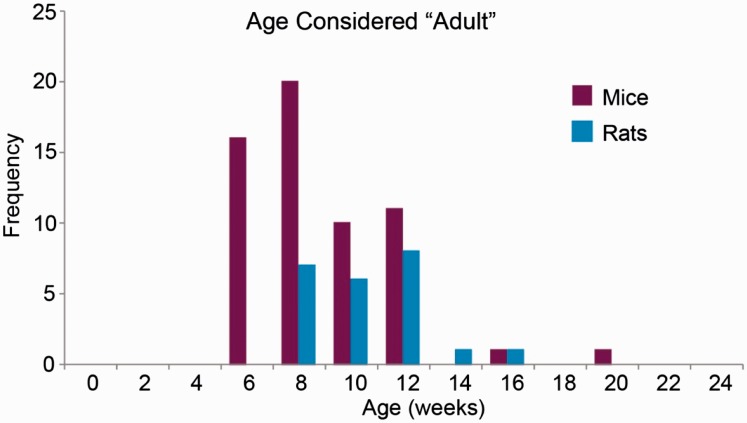
Age considered adult: models described as ‘adult’ were identified, and the ages plotted as frequencies. This illustrates that the description ‘adult’ can encompass a wide range of ages, between six and 20 weeks for mice and between eight and 16 weeks for rats. These ages encompass ongoing development in a range of systems which could adversely affect the outcomes of an experiment. This could be avoided by basing the choice of age on the development of the system under examination. In addition, precise ages should be reported rather than ambiguous terminology such as ‘adult’.

Responses (11.5%) cited that the reason for the choice of age was the rodent being ‘adult’. However, the age range over which the term ‘adult’ was applied across these responses was 6–20 weeks (Figure 2). This illustrates the potential discrepancy when using general descriptions of rodent age.

Ten percent of the total responses described the use of rodents over 16 weeks of age (24% of these in rats and 76% in mice). The age in these cases was justified by one of four reasons: onset of the disease in rodent did not occur until more than 16 weeks had passed (39%); the researchers wanted to capture advanced disease time-points including comorbidities which occurred after 16 weeks (33%); the animal was of a sufficient size for surgery to be performed (10%); the model was of a disease of older age in humans (18%).

**Figure 3.**
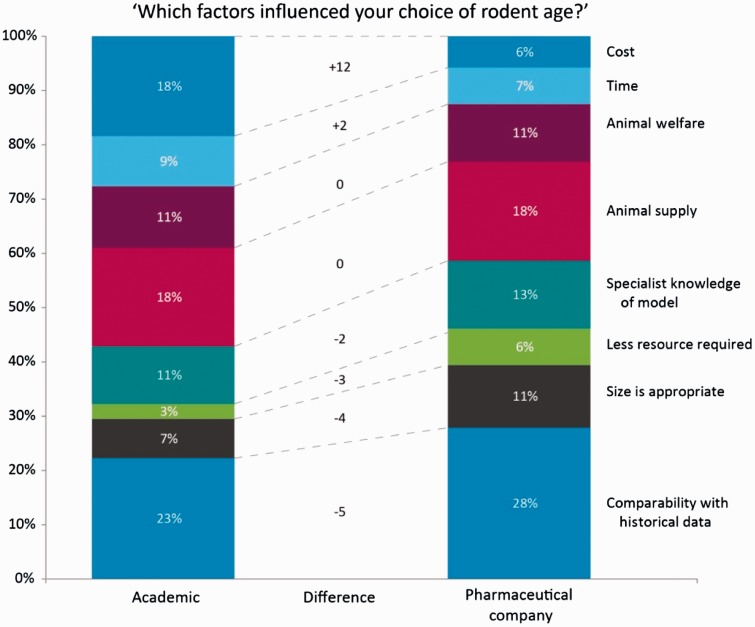
Factors influencing the choice of age of rodent in responses from academic versus industry-based scientists. Respondents were asked to choose reason(s) for their choice of age from a drop-down list. Academic respondents were more concerned with the cost of the animals (18% academic versus 6% industry), whereas industry respondents were slightly more concerned with historical data comparability (23% academic versus 28% industry). This illustrates that ‘practical’ factors can affect age choices, and these differ in academia and industry.

A drop-down list was used to capture the reasons behind the choice of age. Respondents supplied 704 responses, with the highest cited reasons for the choice of age being historical data comparability (24%), supply/availability (18%) and cost (15%). When these reasons were split between academic and industry respondents, a difference was evident (Figure 3). Academic respondents cited the cost of animals as the factor which impacted their choice of rodent age the most (18% academic versus 6% industry), while industry respondents reported that historical data comparability was the most influential factor on the choice of rodent age (28% industry versus 23% academic).

## Discussion

Taken together, these data demonstrate that: (1) choosing an age of rodent relevant to the human disease being studied can have a critical impact on the performance of the model; (2) the age of rodent is often based on practical considerations or convention; and (3) the age of rodent reported as ‘adult’ is inconsistent between studies or laboratories.

### Reporting

Given that reporting of animal age and the reasoning behind the choice of age in publications has generally been poor,^1^ age choices are often based on convention or anecdotal evidence. The NC3Rs’ ARRIVE guidelines^2^ set out the attributes of experimental design to be reported to increase the reproducibility and transparency of in vivo experiments, including the age of animal used. More widespread and stringent adoption of the ARRIVE guidelines will ensure that reporting of animal age is more consistent in the future.

### Consistency in age choice

The majority of responses described using animals between eight and 12 weeks, with a range from two to 120 weeks of age. When the same model paradigm was described in multiple responses, these were compared; the age used varied over a range of up to 20 weeks. This discrepancy in the age of animal used between different laboratories using the same model could lead to data variability obtained between those laboratories. When rodent age is not accurately reported, it cannot be taken into account in subsequent experiments, and this may lead to results not being repeatable elsewhere. This illustrates the importance of using standardized or consensus protocols, and designing experiments with reference to published work on the biology of the system being studied.

### Age considered ‘adult’

By examining responses which indicated that the age used represented an ‘adult’, the disparity when using this terminology was uncovered. The definition of ‘adult’ in this context is likely to be related to the sexual maturity of the rodent. Indeed, rodents are sexually mature and able to breed from around five weeks of age.^40–42^ However, this is not a sufficient basis on which to consider the whole animal to be fully developed. Many systems are immature at this age and may take weeks or months to develop to maturity. Additionally, there may be differences in the maturity of different strains at a particular age.^43,44^ The maturity of the system can significantly impact the outcome of an experiment, as demonstrated in the literature examples cited in this manuscript. ‘Adult’ would be defined differently in many of these examples; therefore this or other terminology referring to a ‘stage of life’ should be replaced with the actual age used when designing, documenting and reporting an experiment.

Where animals over 16 weeks of age were detailed in the response, the range of reasons used to justify this were very narrow, either pertaining to the biology of the model animal or the human disease being modelled. This reflects the fact that older animals are only likely to be used when justified by a specific biological attribute related to ageing; this principle is rarely applied to younger rodents.

### Reasons for age choice

By gathering qualitative data regarding reasons for age choice for a large range of rodent models, we have demonstrated that ‘cost’ and ‘supply’ are second only to ‘historical data compatibility’ as reasons for choosing the age of rodent for a study. The cost pressure is particularly evident when academic respondents are analysed separately from pharmaceutical company respondents. Several academic respondents commented that cost precluded modelling in aged animals and that including higher costs for aged animals would appear unattractive on a grant application. Comparison of the cost of animals at six and 12 weeks from one major commercial supplier to the UK and US markets illustrates this issue: purchase of animals at 12 weeks will result in a cost increase of at least 50% per animal, sometimes close to 100% (Table 1). Researchers who purchase young animals and age them in-house will also face increased costs for housing and husbandry during this period. In order to support more robust and predictive rodent studies, funding bodies and researchers will need to work together to develop a strategy which takes into account that use of older rodents may be beneficial to data quality and the appropriate use of animals, and where justified assess the increased animal costs on grant applications accordingly.

**Table 1.** Percentage increase in cost of 12-week-old rodents compared with 6-week-old rodents.

	C57BL/6J	BALB/c	SD	Wistar
Commercial UK supplier	57	91	69	98
Commercial US supplier	82	68	95	76

Source: a commercial laboratory animal supplier (correct as of August 2015). SD: Sprague-Dawley.

### Comparisons between mice and humans

Some respondents to the survey indicated that they were using a certain age of rodent as it was an equivalent age to the human being modelled. Equivalent ages between rodent and human have been defined by comparing survival rates between mice and humans over their lifespans. For example, one study^41^ defined a mature adult C57BL6/J mouse as 3–6 months (equivalent in this analysis to 20–30 human years), a middle-aged mouse as 10–14 months (equivalent to 38–47 human years) and an old mouse as 18–24 months (equivalent to 56–69 human years). The mature adult mouse represents a stage where development has ceased, but senescence has not yet started, and is recommended as the comparative age for studies into the effects of ageing. Using this approach, 12 weeks should be the minimum for a model of adult disease. Similarly calculations to ascertain equivalent rat and human ages have been published which illustrate the discrepancy in the speed of development at different stages between rats and humans.^40,42^ A framework and online tool have also been developed to translate neurobiological development across multiple species, including rodents and humans.^45,46^

### Databases and information sharing

The Mouse Phenome Database^47,48^ (Jackson Laboratory, Bar Harbor, ME, USA) was the most frequently cited resource in the questionnaire for assessing the appropriate age for mice. Other resources cited include the Age-Phenome Knowledgebase^49^ (Ben Gurion University of the Negev, Beer Sheva, Israel) which allows access to literature sources based on searches of age, phenotype, or both, in mice and humans. Similar resources for rats are not available, but the Rat Genome Database (Bioinformatics Research Center, Milwaukee, WI, USA) is intended to encompass these data in the future.^50^

To facilitate studies on aged rodents and reduce the overall numbers of animals used, networks such as the Shared Ageing Research Models (ShARM) initiative^51^ aim to promote the use and knowledge of aged rodents as models of ageing. In the US, the Aging Rodent Colonies project at the National Institute on Aging (Bethesda, MD, USA) banks and breeds aged rodents and tissues for supply to National Institutes of Health (NIH)-funded researchers working on ageing.^52^

## Conclusion and recommendations for best practice

Use of an inconsistent age of rodents in many areas of scientific research may impact data quality. By increasing awareness of this basic experimental parameter, it may be possible to improve model reliability and reduce the number of animals used in some experiments. Use of an appropriate and consistent age choice plus an improvement in reporting of age will require engagement of researchers across the academic and industry communities. Importantly, given the cost implications of using older animals, engagement of funding bodies is required to ensure that this is taken into account in research funding applications.

### Recommendations

- The age of rodent used in a study should be based on the development or cellular ageing of the system or disease under scrutiny.
- Funding bodies and grant reviewers may need to take into account the increased costs associated with using older animals, and application for increased funding for this should be considered where justified.
- Effective communication between researchers and animal facility staff/veterinarians will increase the likelihood that an appropriate age is chosen.
- Accurate and consistent reporting of age in peer-reviewed literature will allow comparison of experimental methodology and development of consensus-based best practice.

## Supplementary Material

Supplementary material
