# Supplementary material for: Does age matter? The impact of rodent age on study outcomes
Source: Lab Anim. 2016 Jun 15;51(2):160–9. doi: 10.1177/0023677216653984 (PMC5367550; doi:10.1177/0023677216653984)
Supplement: Supplementary material [file LAN653984_supplementary_material.pdf]

Supplementary Data

Supplemental case study one - Immune system maturity affects variability in an acute intravenous lipopolysaccharide (LPS) model in mice

In this study conducted within the Immunoinflammation Therapy Area Unit at GlaxoSmithKline, Stevenage, UK, the immune response to lipopolysaccharide (LPS) in 5-9 week old mice was examined by measuring production of interleukin 6 (IL-6). Data shown represents the vehicle control arm of the original experiment only.

Methods: Case study 1 includes previously published data from a pharmaceutical company laboratory. No additional or repeat animal experiments were carried out to produce this data. All animal studies were ethically reviewed and carried out in accordance with UK's Animals (Scientific Procedures) Act 1986 and the GlaxoSmithKline Policy on the Care, Welfare and Treatment of Animals. Data shown represents the vehicle control arm of the original exploratory experiments only. Data was pooled retrospectively from several studies to achieve group sizes shown (5-6 weeks: n=7; 6-7 weeks n=10; 7-8 weeks n=7; 8-9 weeks n=5; total n=29). Male BALB/c mice were singly housed, maintained at 21.0±2 °C, 55%±10% humidity and fed on a maintenance diet (5LF2 EU Rodent Diet 14% supplied by PMI Labdiet, Richmond, Indiana, USA), had free access to water and were kept on a 12 hour light/dark cycle. Animals aged 5-9 weeks were randomised into groups and intravenous (IV) administered with vehicle followed by IV injection of 1 mg/kg LPS 15 minutes later. 90 minutes-post LPS injection, animals were terminally anaesthetised and cardiac blood was obtained. The blood was allowed to clot, centrifuged at 1000 x g and the resulting serum analysed for IL-6 content using the Mesoscale Discovery platform. Data were analysed by calculating the standard deviation from the mean for each group.

Results: Differences in variability were evident in IL-6 between vehicle control groups of different ages, with the younger (5-6 week) age group displaying high variability (group average 33.4 standard deviation 18.9) compared with older (8-9 week) animals (group average 12.0 standard deviation 4.7; **Fig. S2**). The standard deviation within each group shows a stepwise reduction with age.

Conclusions: This model showed reduction of variability in groups containing older animals. This reduction is possibly due to increased immune system maturity having been reached in the older mice. The greater power derived from lower variability gives higher confidence in the data generated and the impetus to reduce group sizes. Decreasing the variability in these models has been significant as it has had a substantial effect on decision making processes with regard to progression of compounds

Supplemental case study two - Effects of age on the murine mucosal reflex

In this study, a series of *ex-vivo* experiments were carried out to examine whether changes in the murine colonic mucosal reflex could explain previously published data showing that colonic motility was reduced with age. Excised colon was studied at 3, 12, 18 and 24 months of age. Mucosal reflexes were evoked by stroking the mucosa with a fine paint brush and changes in circular muscle tension were recorded using isometric force transducers attached to the distal colon.

Methods: Case study 1 is an analysis of unpublished data from previous studies in an academic laboratory. No additional or repeat animal experiments were carried out to produce this data. All animal experiments conformed to UK's Animal (Scientific Procedures) Act 1986 and were approved by the University of Brighton Ethics Committee. Male C57BL/6J mice were obtained from Harlan UK at eight weeks of age and housed in groups of three to four, under barrier-reared conditions until required. Animals were maintained at 19.0 ± 1 °C, 55 % humidity and fed on a maintenance diet (irradiated RM1 (E) 801002 chow, Special Diet Services) and had free access to irradiated water. The animals were kept on a 12 hour light/dark cycle and studied at three, 12, 18 and 24 months of age. 24 animals were used in total, calculated based on previous experience with the model.

Animals were euthanised by carbon dioxide overdose followed by cervical dislocation. The whole colon was harvested from three, 12, 18 and 24 month old animals (n=6 per group) and placed in ice cold Krebs buffer solution. The colon was opened along its mesenteric border using fine scissors and pinned mucosal surface up in a Sylgard-lined flow bath and perfused with oxygenated Krebs buffer solution at 37 ± 1 °C at a flow rate of eight ml min-1. Mucosal reflexes were evoked by stroking the mucosa with a fine paint brush (2/0) at a rate of one stroke per second and changes in circular muscle tension recorded using isometric force transducers attached to the distal colon. Stroke response curves were plotted for each age group. The area under each evoked response was calculated and differences examined using a repeated measures two-way ANOVA. Data were analysed using repeated measures 2-way ANOVA (GraphPad Prism). Data represent the mean ± SEM.

Results: Greater variability in circular muscle tension was evident in the evoked mucosal responses in 3 month colon compared to the 12, 18 and 24 month groups (**Fig. S3**). Increasing the number of brush strokes failed to significantly change the evoked responses in 3, 18 and 24 month colon. However, a significant stroke response relationship was observed in the 12 month colon.

Conclusions: The greater variability in the three month colon compared to the older age groups, coupled with the significant stroke-response relationship in the 12 month age group, is strongly suggestive that the function of the mucosal reflex matures between 3 and 12 months. In the 18 and 24 month age groups the mucosal response was significantly reduced and the stroke-response relationship is lost. This may explain some of the inconsistency in published data using this model [34-37]. Using animals of an appropriate age allows for a fully functioning reflex to be studied and the reduced variability in the data leads to a reduction of group sizes through increased power.

Figure S1 - Data Analysis Workflow: Illustration of the handling of data-points from the survey and criteria for exclusion. Models were discarded if they did not contain any data about age, did not contain sufficient information to classify by system/process and disease/condition, did not contain a justification for the age used, and when this could not be verified from literature sources.

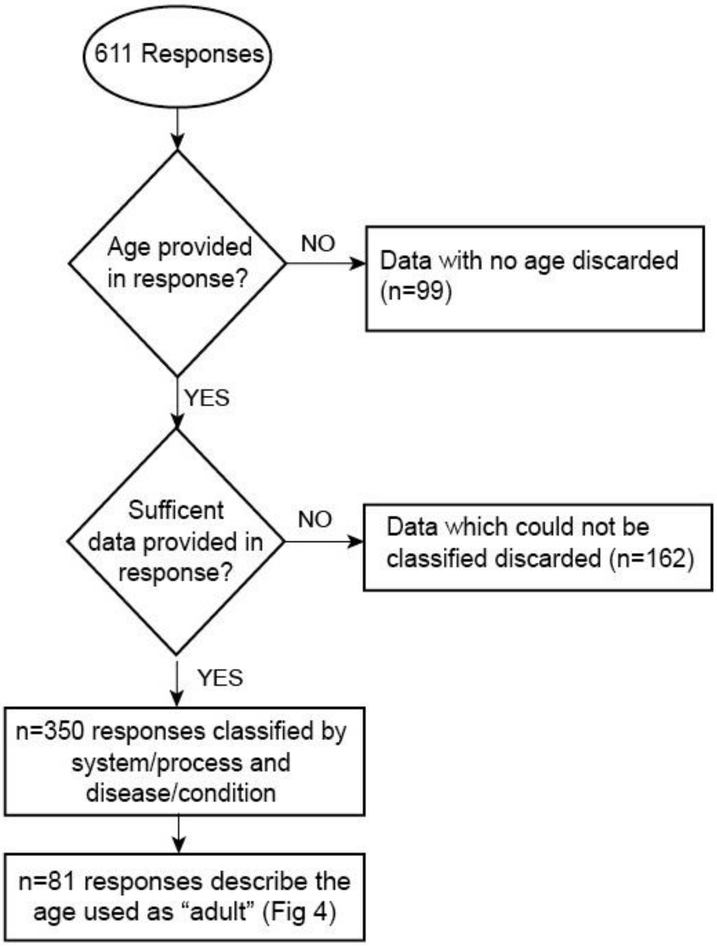

Figure S2 - Age effect on variability, mouse lipopolysaccharide model: male BALB/c mice were injected with lipopolysaccharide, and serum IL6 levels were measured by ELISA. In mice of differing ages (5-9 weeks), significant differences were seen in the variability of response within each group, with younger mice showing a more highly variable response to lipopolysaccharide. This was illustrated using standard deviation from the mean for each group, which decreases with increasing age.

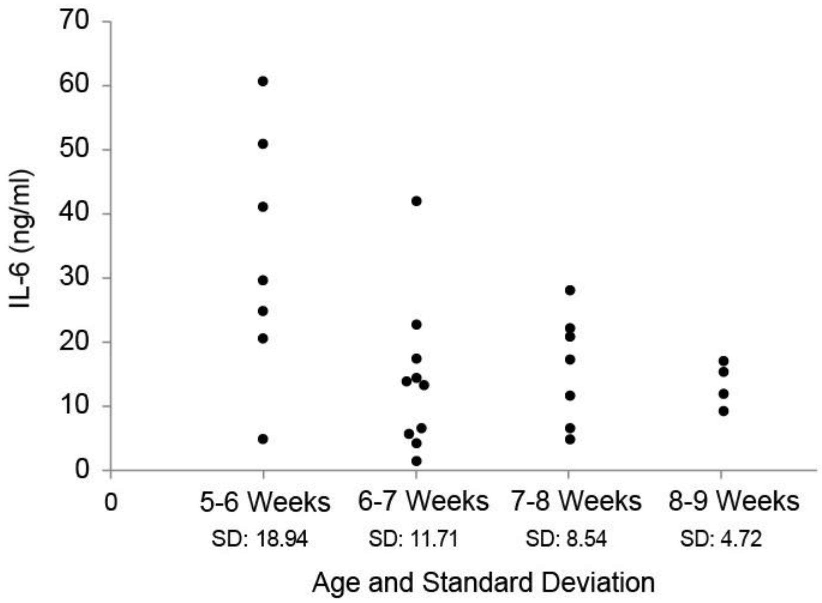

Figure S3 - Effects of age and brush stroke on the area of the mucosal reflex. Excised murine colonic mucosa from male C57BL/6J mice was stimulated to induce a reflex, producing mucosal circular muscle contraction changes. These changes were found to differ significantly between mucosa from 3, 12, 18 and 24 month-old mice, indicating that mucosal responses are age-dependant and do not mature until between 3 and 12 months of age. ** p<0.01; ***p<0.001 *versus* 3 month control; # p<0.05; ##p<0.01 *versus* 12 month; n=6 per data point.

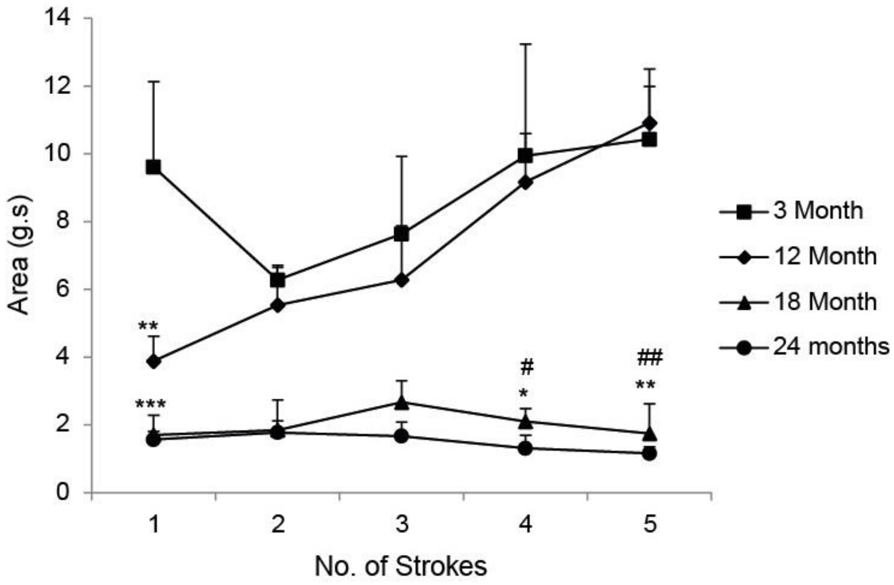

Table S1: Questionnaire to assess age of use of rodents for laboratory models

Q1: Please specify the type of organisation in which you currently carry out your research	▪ Academic<br>▪ Pharmaceutical company<br>▪ Chemical company<br>▪ Biotechnology company<br>▪ Contract research organisation<br>▪ Small business enterprise<br>▪ Animal Breeder<br>▪ Other (please specify)	Drop-down 1 answer allowed from those listed
Q2: Please choose the area of research your currently work in	▪ Neuroscience<br>▪ Oncology<br>▪ Respiratory Disease<br>▪ Pharmacology<br>▪ Toxicology<br>▪ Immunology/Inflammation<br>▪ Laboratory Animal Sciences<br>▪ Tissue Engineering/Regen. Med.<br>▪ Pain<br>▪ Cardiovascular<br>▪ Animal welfare<br>▪ Aging<br>▪ Nutrition<br>▪ Metabolic disease<br>▪ Musculoskeletal disease<br>▪ Opthalmology	Drop-down 1 answer allowed from those listed
Q3: Which rodent models do you use most in your lab? (Space to enter data for up to 4 models)	▪ Rodent model<br>▪ Age (weeks) and/or Weight (g)<br>▪ Strain<br>▪ Species<br>▪ What is your reasoning for using this age?<br>▪ Other comments	Text box<br>Text box<br>Text box<br>Text box<br>Text box<br>Text box
Q4: Which of the following, if any, are additional factors outside of scientific rationale that impact on choice of age of rodent used in a study?	▪ Supply/Availability<br>▪ Less resource required (eg compound)<br>▪ Animal Welfare<br>▪ Cost<br>▪ Time Pressure<br>▪ Specialist knowledge of age-related changes<br>▪ Historical data comparability<br>▪ Size impacting blood volume/tissue or organ size	Drop-down Unlimited answers allowed

Table S2: “System/process” Classification (Wellcome Trust)

Ageing	Immune System
Biomolecular structures / organisation	Lifestyle / human population studies
Breeding	Membrane function
Cardiovascular	Metabolism
Cellular processes	Musculoskeletal
Developmental systems	Nervous system / Mental health
Drug and Vaccine development	Renal / urinary system
Endocrine	Reproductive system / reproductive health
Gastrointestinal / digestive system	Respiratory
Haematopoietic system	Skin and hair
Hearing	Transgenics
Heredity	Vision
